# Current controversies in acromegaly care

**DOI:** 10.1210/clinem/dgag156

**Published:** 2026-04-15

**Authors:** Joanna L Spencer-Segal, Lisa B Nachtigall

**Affiliations:** Department of Internal Medicine, Division of Metabolism, Endocrinology and Diabetes and Michigan Neuroscience Institute, University of Michigan, Ann Arbor, MI 48109, USA; Neuroendocrine and Pituitary Tumor Clinical Center, Massachusetts General Hospital, Boston, MA 02114, USA; Harvard Medical School, Boston, MA 02115, USA

**Keywords:** acromegaly, IGF-I, somatostatin receptor ligand, pregnancy, facial recognition, diagnostic delay

## Abstract

Persistent disease after surgery for acromegaly is common. Expanding medical management options improves long-term biochemical and clinical outcomes, but controversies in acromegaly care remain. We review literature and provide recommendations regarding accelerating diagnosis, biochemical monitoring, symptom variability, preconception/pregnancy management, predictors of treatment response, and alternative dosing regimens. Delayed diagnosis of acromegaly persists, associated with increased morbidity. Advances in artificial intelligence may reduce diagnostic delay; but ethics, privacy issues, and application in diverse populations present challenges. Symptom variability is observed in patients receiving long-acting somatostatin receptor ligands (SRLs) and with other regimens, even when biochemical control is achieved. Dose titration intervals vary across therapies; discordance between biochemical and clinical response requires consideration of signs and symptoms in addition to insulin-like growth factor 1 levels. Premenopausal women with uncontrolled acromegaly are at risk of increased complications during pregnancy. Data on fertility, pregnancy, and fetal effects of acromegaly drugs are limited to case reports and series. Pregnancies and spontaneous conceptions occur in patients with acromegaly, typically with good maternal and fetal outcomes. Predictive algorithms identifying for whom first-line SRLs will be effective have been validated in investigational contexts, but implementation strategy, accuracy, and standardization in clinic practice are yet undetermined. Finally, alternative dosing regimens show promise for decreasing medication burden, cost, and/or improving control. We conclude that while there has been progress in emerging medical treatment options, controversies in acromegaly care persist, particularly with regard to patient-centered outcomes and utility of personalized approaches in real-life, large-scale clinical practice.

## Accelerating diagnosis times from onset of symptoms

**Figure 1:**
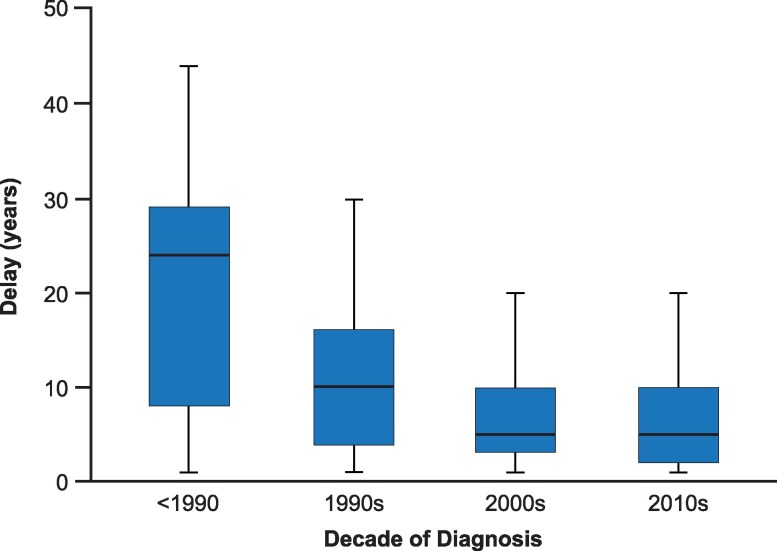
Estimated delay between the first symptoms of acromegaly as reported by patients and the diagnosis of acromegaly and displayed by the decade of diagnosis. Reprinted from Petrossians et al (7) under Creative Commons Attribution 3.0 Unported License, https://creativecommons.org/licenses/3.0/.

A critical unmet need in acromegaly care is decreasing the time from the first signs and symptoms to diagnosis. The delay in diagnosis was reported as being as long as 10 to 20 years in 1962 (1) with a mean delay of 9.2 years in 1987 (Table 1) (2). However, the delay decreased somewhat after 2000 (Fig. 1) (7). Furthermore, studies published before 2000 report a mean delay of 6.6 years to >20 years (1-4), compared with after 2000 in which reported mean times to diagnosis were 3.2 years to 5.5 years (Table 1) (5-9). Yet, ironically, although the classic signs and symptoms are quite visible once the disease is recognized, acromegaly still typically takes ∼5 years to diagnose and more than 10 years in a significant subset of patients (6, 8, 9). In addition, the range in any given cohort is fairly large; for example, in a Swedish population, 65% were diagnosed within 4 years, but 18% required >10 and up to >30 years to be diagnosed (9), suggesting suboptimal progress in establishing the diagnosis at an earlier stage (Fig. 1). The heterogeneity in these reports may reflect variable methodologies of assessment, as some studies rely on record review of subjective and variable patient recall of first signs or symptoms (2, 5-7). Other studies corroborate this with direct surveys (9) or photographs (3) on time since first acromegaly comorbidity was registered (8). It takes longer (2 years) for females to achieve a diagnosis than males (7, 9), indicating the need for improved awareness of acromegaly, particularly in women.

**Table 1:** Time from disease onset to diagnosis of acromegaly reported over 7 decades

Year of publication	Year of diagnosis	Number of patients	Mean years disease onset to diagnosis	Median years disease onset to diagnosis	Method of determining time of onset	Region
1962 Gordon et al (1)	Before 1940-1960	100	10-20	NA	Not specified-record review	Toronto, Canada
1980 Alexander et al (3)	1930-1970	164	6.6	3.7	Patient and relative recall and photographs for 127	Newcastle, UK
1987 Nabarro et al (2)	1963-1983	256	9.2	7	Patient recall of first sign or symptom	London, UK
1988 Bengtsson et al (4)	1955-1984	153	10.2	NA	Not specified Record review	Sweden
2008 Nachtigall et al (5)	1985-2005	100	3.2	NA	Patient recall of first sign or symptom	Boston, MA, US
2010 Reid et al (6)	1981-1994 1995-2005	324	5.9 5.2	NA	Patient recall of first sign or symptom	New York, NY, US
2017 Petrossians et al (7)	<1990 1990s 2000s 2010s	3173		>20 10 5 5*^b^*	Patient recall of first sign or symptom	14 European centers (Liege, Belgium*^a^*)
2020 Esposito et al (8)	2001-2013	603	5.5	3.3	First diagnosed comorbidity recorded	Sweden
2025 Forsgren et al (9)	1991-2017	133		4	Survey and record review	Sweden

Abbreviations: IQR, interquartile range; NA, not applicable; UK, United Kingdom; US, United States.

^*a*^Region of primary authors.

^*b*^Overall, the median delay in diagnosis was significantly longer for females (10 years [IQR: 4.0-18.0]) vs males (8 years [IQR: 4.0-15.0]); *P* = .01.

Potential causes for a longer diagnostic delay in women were not specifically evaluated in the aforementioned studies (7, 9), but the finding that that older women present with milder disease (7) provides a plausible explanation since milder symptoms may take longer to recognize. Additional possible reasons for longer interval between symptom onset and diagnosis in women may result from overlap with other common medical issues in women confounding the diagnosis, such as perimenopause, arthritis, and polycystic ovary disease (10). Effects of endogenous estradiol on GH secretory patterns (11), and lower insulin-like growth factor 1 (IGF-I) levels in women with acromegaly (12), especially in women receiving exogenous estrogen (13, 14), may also contribute. From a health system perspective, provider biases may result in a different approach to complaints in women compared with men (15).

**Figure 2:**
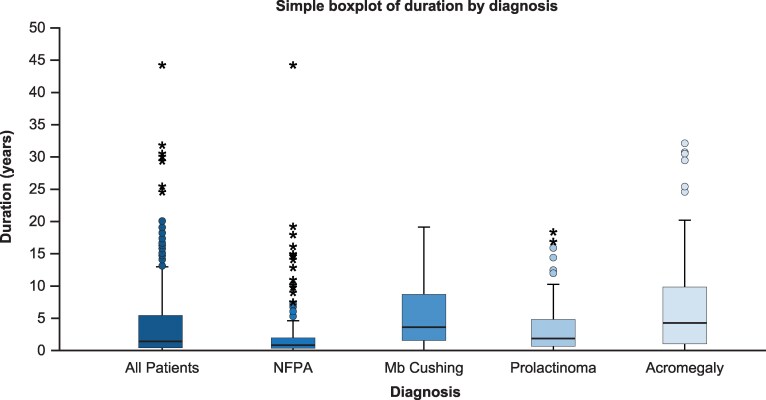
Duration from first symptom to diagnosis in 535 patients with pituitary adenoma. Reprinted from Forsgren et al (9) with permission from BMJ Publishing Group. Abbreviations: Mb Cushing, adrenocorticotropic hormone (ACTH)-secreting adenoma; NFPA, nonfunctioning pituitary adenoma.

The delay in the diagnosis of acromegaly is longer than those for Cushing Disease, prolactinoma, and nonfunctioning pituitary adenoma (Fig. 2) (9). The reasons why acromegaly has traditionally been under-recognized for many years include slow progression, making the change less obvious to the patient and clinician; overlap of some comorbidities with common disorders; the rare occurrence limiting familiarity encountering it; and the variety of organ systems involved that typically direct the patient to multiple different subspecialists, who focus on 1 specific issue (eg, sleep apnea, carpal tunnel, or colonic polyps) but fail to integrate the constellation of problems (16). Earlier diagnosis is important to improve outcomes with higher surgical remission and fewer adverse comorbidities and less mortality (8, 17).

Attempts to decrease the time to diagnosis have included creating a simple targeted screening survey and implementing it in ambulatory care (18). In 17 000 patients in a primary care clinic, 2 questions were asked: “Has your shoe size increased over the last 5 years?” and “Did you have to change your wedding ring or ring over the last 5 years because it became tight?” If a patient answered yes to at least one of these, IGF-I level was measured (18). One patient was identified based on clinical signs and symptoms suspected by the clinician, but an additional 5 patients were identified from the subgroup of 178 patients who answered “yes” to at least 1 question. This approach increased acromegaly prevalence by a factor of 5, suggesting that active screening increased case finding (18). However, only 2.8% (5/178) of those identified for screening with IGF-I measurement had confirmed IGF-I excess (18). Other screening tools to enhance earlier diagnosis, such as ACROSCORE, which incorporates diabetes, carpal tunnel syndrome, and sweating, have been tested in patients with known acromegaly but not in the general population (19). Clinical patterns derived from data mining of insurance databases to facilitate earlier detection are of limited utility if not verified by actual chart review (20).

Facial recognition programs have progressed in technical accuracy (21-30), sometimes exceeding the diagnostic accuracy of clinicians (25, 26, 28, 29). A recent facial recognition study shows the promise of earlier recognition with accuracy in identification of facial images from a mean of 7.47 years before diagnosis (21). The 2 earliest studies in 2011 report accuracy of <90% (28, 29) vs the many later reports in which accuracy was >90% (21-27). Most studies include photographs from a homogenous population and may not be applicable across a range of ethnic diversity. For example, a Chinese cohort was used in a large study (26) and another study included mostly White patients (22); privacy issues must be considered before widespread implementation of such software. Unfortunately, while the technical ability to use software to diagnose acromegaly accurately at earlier stages has evolved, issues of privacy, ethics, and application in diverse ethnic populations remain challenges.

Voice recognition software shows promise for the use of acoustic analysis to diagnose acromegaly from a voice recording (31). Patients with acromegaly may develop hoarseness and deepened voice quality due to oral and laryngeal effects of long-term growth hormone (GH) excess, effects on vocal tract, and increased vocal fold volume (31, 32). Benefits of digital voice recognition software include convenient access to voice recordings from mobile phone data and fewer privacy concerns than facial recognition. However, this was a homogeneous population in Sweden with a single language, and neck surgery, asthma, upper respiratory infection, or neck radiation, representing approximately 25% of the volunteers with acromegaly, were excluded, but in the real-world this may confound results.

A deep learning model using a convoluted neural network detected the disease from hand photographs and demonstrated high performance, with a sensitivity of 98% and a specificity of 92% (33). Applying artificial intelligence and machine learning using hand rather than facial images to detect acromegaly decreases the risk of privacy invasion and may allow for earlier disease diagnosis, particularly in patients who do not display classic facial changes (34).

## Biochemical monitoring recommendations (including timing of IGF-I measurements during depot SRL injection therapy)

### When to check IGF-I in patients receiving long-acting injectable SRLs

Consensus guidelines have suggested measuring IGF-I after the first 3 monthly doses in patients with acromegaly receiving long-acting injectable SRLs including octreotide, lanreotide, and pasireotide, and that timing of further follow-up IGF-I assessment depends on the rate and degree of IGF-I control (35). Initial studies on octreotide long-acting release (LAR) suggested long-term stability of steady-state concentrations of the drug over at least 28 days, but the timing of assessment in relation to the time of the last dose, within the month, in patients receiving monthly octreotide LAR, has not been formerly considered until recently (36). Most studies of SRLs do not report drug concentration, but individual variation was noted in peak concentration reached at a given dose (37). Variation in IGF-I levels, with higher levels toward the end of the month (38), provided evidence supporting a “preference” to ascertain IGF-I at the longest interval since the last injection (35). A 2025 consensus update indicated that the timing of IGF-I assessment between injections should be obtained, “preferably immediately before the ensuing one” (35).

**Figure 3:**
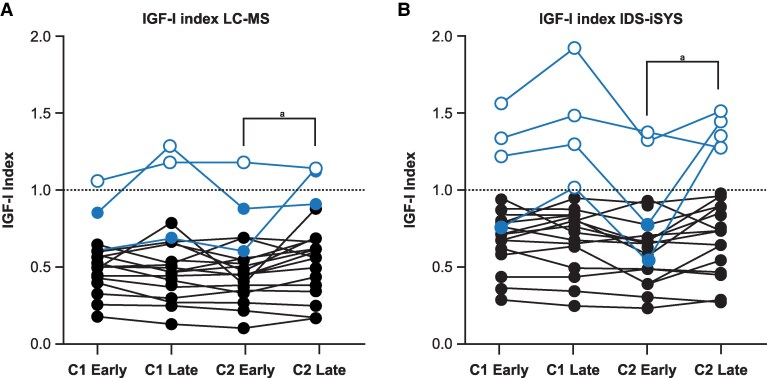
IGF-I level fluctuation according to time since injection in patients with acromegaly receiving therapy with monthly SRL therapy. Injection phase and IGF-I level fluctuation. IGF-I level fluctuation during early and late phases in patients with acromegaly controlled with monthly SRL injections across 2 cycles is shown. The dotted line and gray background indicate the area below the ULN. IGF-I is expressed as IGF-I index (IGF-I/ULN for each assay used). Highlighted in blue are the patients who have at least 1 IGF-I value > 1.0× ULN. Empty circles correspond to IGF-I levels above the ULN at any given time point. Filled circles represent measurements below the ULN. (A) IGF-I index liquid chromatography–mass spectrometry (LC-MS). (B) IGF-I index immunoassay immuno diagnostic system (IDS-iSYS). ^a^*P* < .05. Reprinted from Remba-Shapiro et al (39) with permission from Oxford University Press on behalf of the European Society of Endocrinology. Abbreviations: C, cycle; IDS-iSYS, immuno diagnostic system; IGF-I, insulin-like growth factor 1; LC-MS, liquid chromatography–mass spectrometry; SRL, somatostatin receptor ligand; ULN, upper limit of normal.

IGF-I may vary according to time since last injection (39), revealing a higher serum IGF-I level 26 to 28 days after the last SRL injection, compared with the first week after injection (39). SRL concentrations decline in a subset of patients toward the end of the month, and some patients demonstrate a normal IGF-I 5 to 7 days after injection, with an elevated IGF-I 26 to 28 days after the injection, while on the same dose and drug (39). Thus, timing of the evaluation in relation to the SRL injection schedule may affect the IGF-I observed over the course of 2 months, and measured by both high-resolution liquid chromatography–mass spectrometry (LC-MS) as well as by an automated chemiluminescence assay (39). In both assays, several patients thought to be controlled would be classified differently if the assessment was done toward the end of the month interval between injections (Fig. 3) (39).

Oral glucose tolerance testing (OGTT) to assess GH suppression in patients treated with SRLs is not useful (40). Initial and dose-adjusted IGF-I assessment is best done approximately 12 weeks after injection, with less frequent monitoring, typically every 12 months, for patients on stable maintenance therapy (35, 41-43). Insulin-like growth factor 1 and GH do not always correlate and, therefore, occasional testing of GH is indicated, particularly with discrepant results or with adenoma growth despite normal IGF-I (44-46). In patients receiving pasireotide, baseline hemoglobin A1C and fasting blood glucose should be tested and repeated with weekly fasting blood sugars in the first 3 months and 4 to 6 weeks after a dose change due to the risk of hyperglycemia associated with initiation of this drug (47, 48). Periodic testing of hemoglobin A1C should continue with long-term pasireotide therapy (47).

### When to check IGF-I levels in patients receiving oral SRLs

Initial IGF-I monitoring is recommended 2 to 4 weeks after starting or changing the dose of paltusotine. Dose titration decisions are based on IGF-I levels at this interval, as shown in a phase 3 clinical trial demonstrating rapid IGF-I normalization and sustained biochemical control within this time frame (49). Thus, IGF-I is checked approximately every 2 to 4 weeks after initiation or titration to assess treatment response and direct further dosing changes. After achieving a stable maintenance dose, monitoring frequency can be adjusted as required. When adjusting the dose of oral octreotide, IGF-I should be checked every 2 to 4 weeks during the dose titration period (50-52). Dose adjustments should be based on IGF-I levels as well as clinical signs and symptoms, with a starting dose of 40 mg per day and maximum recommended dose of 80 mg per day. Once a maintenance dose is established, IGF-I monitoring is generally recommended according to the patient's stability and the clinician's discretion (50-53). Insulin-like growth factor 1 could be reassessed if proton pump inhibitors, H2-receptor antagonists, or antacids are initiated, as these may decrease oral octreotide bioavailability (51).

### When to check IGF-I in patients receiving a dopamine agonist or pegvisomant

Cabergoline or pegvisomant requires monitoring of IGF-I monthly after initiation of the drug or a dose change and then as clinically indicated (35, 43, 54). Measuring GH is not useful in patients receiving pegvisomant, as it blocks GH action and GH levels may increase due to decreased IGF-I feedback, even when patients are well controlled clinically and IGF-I is normal (35, 43, 55).

## Impact and control of symptom variability

**Figure 4:**
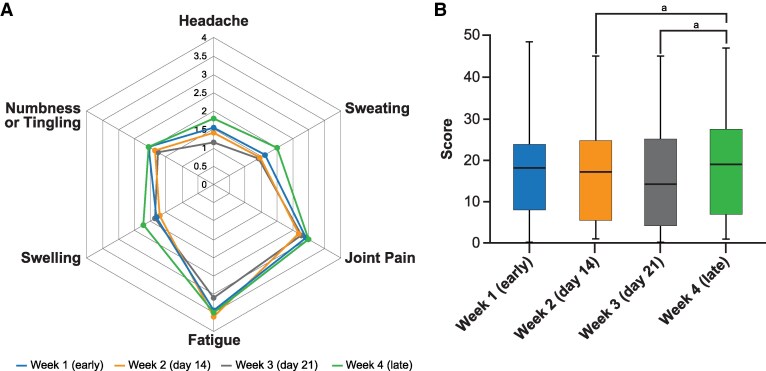
Patient-assessed acromegaly symptom questionnaire (PASQ) according to time since somatostatin receptor ligand injection. (A) Each value represents the pooled weekly PASQ score average for each symptom from the 2 injection cycles. Each symptom is graded on a scale from 0 to 8, where 0 indicates absence and 8 represents the highest severity. Week 1 (early phase) is represented in blue, week 2 (day 14) in orange, week 3 (day 21) in gray, and week 4 (late phase) in green. (B) Average of the weekly PASQ total score from the 2 injection cycles (total weekly PASQ score reflects the sum of each symptom). Reprinted from Remba-Shapiro et al (39) with permission from Oxford University Press on behalf of the European Society of Endocrinology. ^a^*P* < .05.

**Figure 5:**
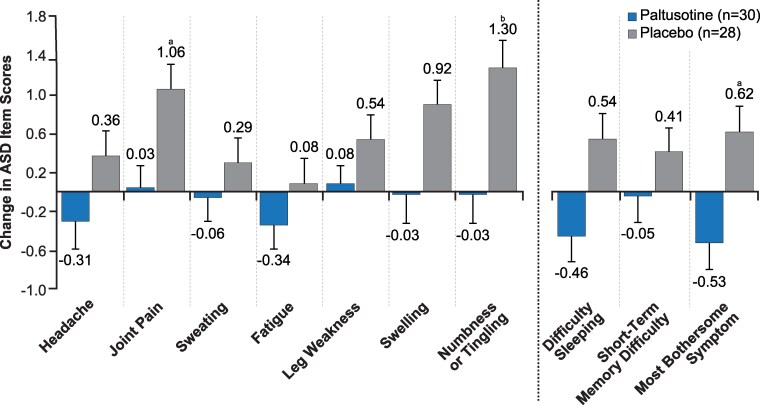
Least-squares mean change in ASD item scores from baseline to end of treatment (36 weeks). Higher scores indicate worsening. Dashed line separates core items (left) from additional items and most bothersome symptom (right). Change from baseline values (reported as least-squares means) and *P* values are from analyses of covariance. Error bars indicate standard error. Adapted from Gadelha et al (64) under Creative Commons Attribution 4.0 License, https://creativecommons.org/licenses/by/4.0/. ^a^*P* < .05 for paltusotine vs placebo; ^b^*P* < .01 for paltusotine vs placebo. Abbreviation: ASD, Acromegaly Symptom Diary.

Symptoms and signs of acromegaly, as well as mortality, improve with control of IGF-I (54, 56, 57). However, patient-reported symptom outcomes may be discrepant with IGF-I control (58-60), particularly in those receiving long-acting SRL injections in the latter part of their SRL injection schedule (Fig. 4) (39). In patients receiving SRLs, symptoms varied according to the injection timing and were overall worse as rated by patients in a survey of symptoms, the Patient-Assessed Acromegaly Symptom Questionnaire (PASQ), in the last week before the next injection was due (Fig. 4) (39). The PASQ, validated for acromegaly-assessed headache, hyperhidrosis, joint pain, fatigue, soft tissue swelling, and numbness or tingling, is graded by patients on a scale from 0 to 8, where 0 indicates none and 8 is the most severity, with an additional item on overall health, graded from 0 to 10, with 0 representing optimal health and 10 indicating the worst health status (39, 61, 62). Individual patterns varied without a consistent correlation between worsening symptoms and rising IGF-I levels; symptoms and IGF-I varied over time in relation to the injection cycle but not always in relation to each other (39). In the randomized trial of paltusotine vs placebo, patient-reported symptoms were assessed using a novel validated acromegaly symptom score, the “acromegaly symptom diary,” which included headache, joint pain, sweating, fatigue, leg weakness, swelling, numbness/tingling, plus additional items for sleep difficulty and short-term memory difficulty (49, 63, 64). Symptom scores were stable or significantly improved throughout the treatment period but rose in the placebo arm (Fig. 5) (49, 64).

## Management of acromegaly during preconception and pregnancy

Because fertility is adversely impacted by GH excess and an expanding sellar mass, pregnancy in patients with acromegaly was once a rare event. Many patients question whether fertility is possible, safe, and likely to have a favorable outcome for both the mother and the fetus. With improved treatments for acromegaly and infertility, the short answer to this question is yes. Here, we review the approach to women with acromegaly who seek fertility.

**Figure 6:**
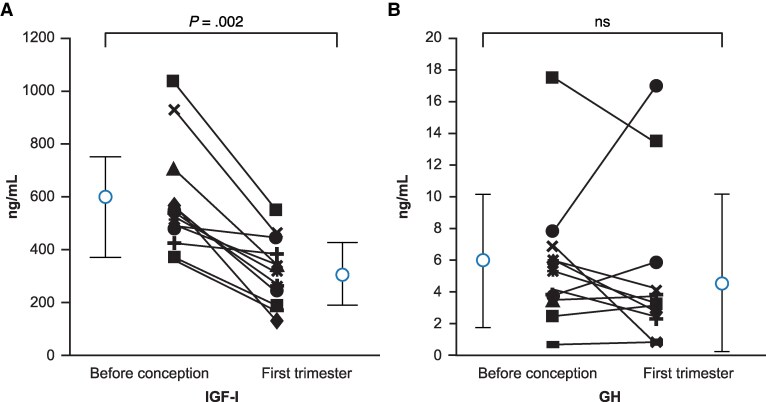
Changes in IGF-I and GH levels during the first trimester of pregnancy in 12 women with acromegaly. First time point before conception, second time point at first trimester. Adapted Caron et al (69) with permission from Oxford University Press on behalf of the Endocrine Society. Abbreviations: GH, growth hormone; IGF-I, insulin-like growth factor 1; ns, nonsignificant.

High rates of menstrual cycle dysfunction, frank hypogonadism, and infertility in premenopausal women with untreated acromegaly are attributed to compressive mass effects, complications of sellar surgery, hyperprolactinemia, and peripheral effects of hyperglycemia and excess GH. However, effective surgical and medical treatments correct menstrual cycle irregularities in most patients, enabling many to achieve a spontaneous pregnancy (65-68). Pregnancy-associated changes in GH and IGF-I physiology in patients with and without acromegaly include lower IGF-I levels during the first trimester (Fig. 6) (69-75). In patients with acromegaly, IGF-I levels may drop by at least 30% in the absence of changes to GH levels, likely due to estrogen inhibiting hepatic GH action (70, 76, 77). In our experience, patients usually also report improved acromegaly symptoms during the first trimester. During the second and third trimesters, there is increased production of both placental GH and placental lactogen, leading to increased IGF-I levels (78). In healthy pregnant women, pituitary GH falls, presumably due to negative feedback from these placental hormones. Therefore, patients with uncontrolled acromegaly at baseline will often have an IGF-I level within the trimester-specific range for healthy controls during their pregnancy (70).

Pituitary-derived GH, placental GH, and IGF-I do not cross the placenta (76), alleviating concerns about adverse effects of acromegaly on the fetus. The greater risk is for metabolic complications of increased GH, including gestational diabetes and hypertension, because these are more prevalent in patients with uncontrolled acromegaly at baseline than they are in the healthy control population (69, 79). These patients should have close obstetric follow-up and be treated promptly for these complications. Overall, maternal and fetal outcomes in acromegaly are generally favorable (67, 69-71, 75). Pregnant patients with the greatest risk of visual field defects due to adenoma mass effect are those with new acromegaly diagnoses during pregnancy (69, 79).

Medical treatments for acromegaly mostly do not have sufficient human data to clearly determine their safety in pregnancy; therefore, they should only be used when the potential risks outweigh the benefits (Table 2) (67-71, 75, 79-84). Most safety data exist for subcutaneous octreotide and octreotide LAR, which were previously assigned US Food and Drug Administration (FDA) Pregnancy Category B. Octreotide does pass into the fetal circulation (85, 86), and some case reports and series have suggested the possibility of delayed fetal growth with octreotide exposure during pregnancy (69, 82, 85). Additional case reports and series are reassuring regarding the safety of octreotide exposure during pregnancy (67, 71, 74, 87-89). While the somatostatin receptors SSTR2 and SSTR5 targeted by octreotide suppress fetal pituitary-derived GH, neonates exposed to octreotide throughout pregnancy have normal IGF-I levels, perhaps reflecting relatively decreased sensitivity of the fetal pituitary to somatostatin (85). Any effect of octreotide on neurodevelopment, in which somatostatin plays an important role, is likely minimized by poor penetration of octreotide through the fetal blood-brain barrier.

**Table 2:** Medical treatment exposures during conception and pregnancy in patients with acromegaly

Article	Pregnancies/patients	Drugs*^a^*	Maternal outcomes	Fetal outcomes	Safety signals
2024 Tönjes et al (67)	47/31	SRL or DA during pregnancy, 6.4% (*n* = 3 pregnancies)	GDM, *n* = 5 PIH or preeclampsia, *n* = 3 No complications in patients with untreated acromegaly, *n* = 3	No preterm births, 87.9% (*n* = 41) of deliveries without complications 3 voluntary terminations	4.3% (*n* = 2) of newborns each had high (> 4200 g) and low (< 2800 g) birthweight
2023 Jiao et al (79)	11/8	BROM during entire pregnancy, *n* = 1	GDM, *n* = 1 PIH, *n* = 1	1 SA at 12 weeks; 1 early termination due to active acromegaly with intense headache;	None
2021 Das et al (80)	14/12 3 with ART	Acromegaly controlled, *n* = 9; uncontrolled, *n* = 5 Before pregnancy: CAB, *n* = 7 SRL, *n* = 2 SRL + CAB, *n* = 1 None, *n* = 4	No GDM PIH, *n* = 2 1 new diagnosis with visual compromise treated with monthly OCT LAR with normalization of vision; healthy full-term delivery without complications 1 new diagnosis treated with CAB; healthy full-term delivery with postpartum hemorrhage In active disease, more patients had normal IGF-I postpartum compared with time of conception	2 fetal losses in patients using ART to conceive No macrosomia or congenital malformations 1 premature delivery at 28 weeks (maternal PIH) In twin gestation, 1 infant died of neonatal sepsis on day 2	None
2020 Guarda et al (81)	4/3	All patients received PEG while trying to conceive; PEG withdrawn immediately before or at pregnancy confirmation	GDM, *n* = 2	No complications	None
2019 Hannon et al (75)	17/12	Acromegaly controlled preconception, *n* = 5; uncontrolled, *n* = 12 Before pregnancy: OCT, *n* = 4/17 CAB, *n* = 10/17 BROM, *n* = 1/17 DA continued in pregnancy, *n* = 5 (1 BROM, 4 CAB); others stopped	No GDM PIH, *n* = 1 Plasma IGF-I normalized during pregnancy, *n* = 9 No clinically relevant increases in tumor size, visual field deterioration, or progressive headache, *n* = 15/17 assessed	15 healthy infants at term; *n* = 11/17 delivered by C-section 1 emergency C-section due to preeclampsia (32 weeks; birth weight 1200 g); no other PIH 1 elective C-section (35 weeks; birth weights 2450 and 2200 g) for twin pregnancy No adverse fetal outcomes in 5 pregnancies with ongoing DA use	Increased rate of C-section
2018 Jallad et al (82)	31/20	Untreated preconception, *n* = 4 SRL before pregnancy, *n* = 4 CAB, *n* = 2 OCT, *n* = 1 OCT + CAB, *n* = 1 SRL during pregnancy, *n* = 11 OCT, *n* = 10 (throughout, *n* = 5; first 5 months, *n* = 5) PAS, *n* = 1 Comorbidities at diagnosis: diabetes 15%, glucose intolerance 25%, hypertension 30%	GDM, *n* = 2 PIH, *n* = 14 (preeclampsia, *n* = 3; eclampsia *n* = 1) 2 new diagnoses underwent transsphenoidal surgery during pregnancy for visual field impairment, 1 had SA after surgery IGF-I normalized sometime during pregnancy, *n* = 23, in 13 cases without treatment	Live births, *n* = 27 SA, *n* = 4 (including patient treated with PAS) Congenital malformations, *n* = 2 Low birthweight with no malformations, *n* = 3 (2 used OCT for 5 months of gestation) Macrosomia, *n* = 1	Low birthweight, *n* = 2 (from 2/5 women who used OCT for 5 months of gestation) Congenital malformations, *n* = 2 (single kidney, *n* = 1; ureteral stenosis, *n* = 1)
2015 van der Lely et al (83)	35/33	PEG exposure during pregnancy: *n* = 27 ME, *n* = 8 PE	GDM, *n* = 3 (1 continued PEG during pregnancy)	Live births, *n* = 18/35 (5 PE) Normal births, *n* = 14 (4 PE, 10 ME [3 with PEG throughout pregnancy], and 4 premature [1 PE]) Ectopic pregnancy, *n* = 1 (ME) SA, *n* = 2 (ME, both with lanreotide coexposure)	2 spontaneous abortions in patients with PEG and lanreotide coexposure during pregnancy
2014 Dias et al (70)	10/8	Before pregnancy: OCT, *n* = 4 OCT/CAB, *n* = 5 OCT/PEG, *n* = 1 Drugs stopped before pregnancy, *n* = 2; treatment withdrawn at pregnancy confirmation, *n* = 6	Preeclampsia, *n* = 1 Headaches without visual abnormalities, *n* = 3 GDM, *n* = 1 No adverse tumor outcomes No changes in pituitary GH, *n* = 5	Full-term deliveries, *n* = 9 Preterm (35 weeks), *n* = 1 due to preeclampsia No fetal malformations All healthy, normal weight and length	None
2010 Caron et al (69)	59/46	Acromegaly controlled, *n* = 23; uncontrolled, *n* = 34 Before or during pregnancy: SRL, *n* = 14 DA, *n* = 25 In most cases, drugs were discontinued when pregnancy was recognized	*Uncontrolled acromegaly:* PIH, *n* = 6 (18%) GDM, *n* = 3 (9%) *Controlled acromegaly*: PIH, *n* = 2 (9%) GDM, *n* = 1 (4%) Mean IGF-I fell substantially during the first trimester, *n* = 12; no changes in GH Tumor volume increased, *n* = 3 (11.1%); stable, *n* = 22 (81.5%); decreased, *n* = 2 (7.4%) 4 patients with visual impairment during pregnancy (3 new diagnoses), 1 treated with CAB	No major fetal malformations 4 premature deliveries in 3 women, including 2 sets of twins and 1 woman with preeclampsia during 2 births Macrosomia, *n* = 2/50 (4%) infants (not in women with GDM)	2 SRL and 2 SRL + DA pregnancies resulted in SGA infant (exposures were < 2 months) Potential increased risk of GDM, PIH, and preeclampsia in uncontrolled patients
2006 Atmaca et al (84)	7/48	BROM withdrawn during pregnancy, *n* = 3 OCT during pregnancy, *n* = 1	GDM controlled with diet, *n* = 2 1 new diagnosis at 29 weeks with apoplexy at 33 weeks and emergency transsphenoidal surgery No worsening of symptoms or tumor growth, *n* = 4	Therapeutic abortion: chronic OCT during pregnancy, *n* = 1; uncontrolled acromegaly, *n* = 1 1 emergency C-section due to pituitary apoplexy Full-term healthy infants, *n* = 4	None
2006 Cozzi et al (71)	7/6	SRL discontinued 3-4 months before conception, *n* = 5; discontinued at pregnancy confirmation, *n* = 2	No GDM, PIH, preeclampsia, or visual compromise Tumor growth, *n* = 1 GH decreased, *n* = 3; remained stable, *n* = 3; increased, *n* = 1 IGF-I decreased (as % ULN) during pregnancy in all patients, despite no treatment	Uneventful pregnancies with healthy infants	None
1998 Herman-Bonert et al (68)	4/4	OCT discontinued 6 months before pregnancy, *n* = 1; discontinued at confirmation of pregnancy, *n* = 3	No reported complications	Healthy full-term infants, *n* = 3; 1 patient 7 months pregnant when article published	None

Abbreviations: ART, assisted reproduction techniques; BROM, bromocriptine; CAB, cabergoline; C-section, Cesarean section; DA, dopamine agonist; GDM, gestational diabetes mellitus; GH, growth hormone; IGF-I, insulin-like growth factor 1; LAR, long-acting release; ME, maternal exposure; OCT, octreotide; PAS, pasireotide; PE, paternal exposure; PEG, pegvisomant; PIH, pregnancy-induced hypertension; SA, spontaneous abortion; SGA, small for gestational age; SRL, somatostatin receptor ligand; ULN, upper limit of normal.

^*a*^Used before pregnancy confirmation or during pregnancy.

The very limited data on pegvisomant exposure during pregnancy are derived from the Pfizer Global Safety Database (27 patients with maternal exposure) and case reports (81, 83, 90). These results suggest the likelihood of a normal pregnancy with no fetal complications in patients exposed to pegvisomant at the time of conception. In most of these patients, pegvisomant was discontinued when pregnancy was confirmed. Two spontaneous abortions occurred with maternal coexposures to pegvisomant and lanreotide (83). Pegvisomant does not substantially pass into the fetal circulation (90). Pegvisomant, like octreotide, was previously rated Pregnancy Category B by the US FDA.

Our practice and recommendation prioritize avoiding fetal medication exposure whenever possible, which usually requires discontinuing medical treatments at least 4 to 5 elimination half-lives before conception. Practically, this requires stopping long-acting injectable therapies at least 4 months before conception. Patients experiencing acromegaly-related infertility should be counseled on the risks and benefits of continuing medical treatment for GH excess while trying to conceive. The increasing availability of short-acting oral SRLs might enable a shorter discontinuation interval before conception attempts. If a patient receiving octreotide LAR becomes pregnant, this medication should typically be discontinued, but these patients should be counseled that the available data do not suggest a significant risk for fetal malformation or adverse pregnancy outcomes with this exposure. In the rare case that sellar mass effect during pregnancy requires intervention, transsphenoidal surgical resection is usually the preferred treatment option, in consultation with the patient and the obstetric team. If surgery is declined or not possible, case reports have documented favorable visual and fetal outcomes in patients treated with octreotide LAR during pregnancy for this reason (87).

Postpartum women with acromegaly can breastfeed successfully (69). The drug labels for SRLs acknowledge that these compounds are to some extent excreted into the milk of lactating rats, though it is not clear to what extent this is true in humans. These compounds are expected to be poorly orally absorbed by the fetus, but safety data are scant. Pegvisomant is poorly excreted in breastmilk, as expected (90). However, the breastmilk of a lactating woman taking pegvisomant had higher growth hormone concentrations than control (healthy) mothers (90), which could be a safety concern. Several cases of successful breastfeeding during maternal treatment with SRLs and pegvisomant have been reported with no reported adverse effects on infant/child development (90-94). Nonetheless, because of the lack of data, our recommendation is to delay the initiation or reinitiation of these drugs, if possible, until breastfeeding is discontinued.

## Predictors of response to somatostatin receptor ligand therapy for acromegaly

While SRLs are the mainstay of medical therapy in patients not cured by or not candidates for surgery, 45% to 70% of patients have an incomplete biochemical response (95). Understanding and using predictors of SRL efficacy may optimize therapy and improve biochemical control. Imaging, biochemical, adenoma, and patient factors are associated with SRL responsiveness (Table 3) (96-113). Lower baseline IGF-I and GH levels, smaller/less invasive adenomas with T2 hypointensity, abundant SSTR2 expression, older age, and dense GH granularity predict a higher likelihood of achieving IGF-I control with treatment. Molecular markers associated with SSTR2 signaling and granulation pattern also have predictive value (112).

**Table 3:** Predictors of favorable biochemical response to octreotide and lanreotide

Patient phenotype	Biochemical	Imaging	Histologic
Older age (96-101) Lower body weight (98, 99)	Lower baseline IGF-I (96, 98); but higher IGF-I at diagnosis predicted > 50% reduction (99, 100) Lower baseline GH (96, 100, 105, 106)	Smaller tumor size (96, 100) Lower Knosp grade (106) T2 hypointensity (99, 101, 107)	Histologic subtype: densely granulated (96, 97, 100-105) Higher SSTR2 expression (101-104, 108-111) Lower e-cadherin expression (112) IHC; tightly associated with granulation pattern (109) Lower Ki-67 index (103, 109, 113)

This table shows a selection of well-established predictors that have been included in prediction models.

Abbreviations: GH, growth hormone; IGF-I, insulin-like growth factor 1; IHC, immunohistochemistry; SSTR2, somatostatin receptor 2.

There are limitations to relying on currently validated predictors to direct initial medical therapy. An 82.4% accuracy was observed using a machine learning model to predict complete SRL response using 14 known predictors (114). Importantly, the negative predictive value was only 78%, suggesting that about 1 in 5 patients for whom the model predicted an incomplete response would actually be fully biochemically controlled (IGF-I index = patient's IGF-1/age- and sex-specific upper limit of normal) with SRL treatment. In the ACROFAST study (115), patients were risk-stratified based on predicted SRL response and assigned prospectively to SRL (*N* = 21), SRL + pegvisomant combination therapy (*N* = 5), or pegvisomant monotherapy (*N* = 6), as compared with standard of care (*N* = 36). At the 12-month follow-up, complete biochemical control rates were 53% in the standard therapy group and 78% in the personalized groups, suggesting that personalized treatment led to, on average, IGF-I normalization 4 months earlier. Although this study shows efficacy of pegvisomant mono- and combination therapy, a subset of patients are likely to be overtreated with unnecessary pegvisomant using this strategy. This may result in increased long-term medication burden, increased cost, and possible missed opportunity for tumor control by medical therapy in misclassified patients.

We initiate SRLs in most candidates for medical therapy. Although predictors can be used to counsel patients regarding the likelihood of an incomplete response, true nonresponse is rare and poorly predicted. Octreotide and lanreotide are reasonable first-line treatment options for almost all patients since they have fewer side effects than pasireotide. On the other hand, hyperglycemia caused by pasireotide is apparently reversible, and pasireotide can be more efficacious in some patients (116). Somatostatin receptor ligands are generally less burdensome than pegvisomant, and they may have beneficial effects on tumor even in partial responders. Because known predictor sets have low negative predictive value and have rarely been studied in real-world clinical practice (104, 115), offering an opportunity to achieve control on SRL monotherapy as first-line therapy can avoid unnecessary treatment escalation. For patients not controlled on SRL monotherapy, combination SRL + pegvisomant is biochemically effective, minimizing pegvisomant dose while also providing adenoma-directed therapy. Additionally, the biochemical efficacy on which predictive models are based does not uniformly correlate with adenoma shrinkage. Furthermore, the clinical utility of prediction models is limited by lack of standardization of pathologic marker intensity or MRI T2 hypointensity. As other authors have highlighted (117), while individual studies might show a strong relationship between SSTR2 expression and biochemical response to SRLs, consistent clinical implementation of this would require standardization of immunohistochemistry staining and scoring protocols across centers.

Importantly, many clinical and histologic predictors of SRL efficacy are redundant. Accordingly, identifying and measuring independent molecular pathways contributing to SRL resistance could improve outcome predictions. For example, Ki-67 scoring predicted SRL response independently of SSTR2A expression, as did aryl hydrocarbon receptor–interacting protein (AIP) expression (103, 110). We anticipate a role for newer molecular markers of treatment response, including somatostatin receptor variants (118), microRNAs (119), and others (113, 120).

An important conclusion derived from studies of individualized treatment is the importance of drug titration and avoidance of treatment inertia. Individual predictive factors and models can be helpful to identify patients likely to require combination therapy. The newer oral SRLs (oral octreotide and paltusotine) can be titrated relatively rapidly, and initial consideration of these in patients with predictors of poor response could accelerate therapy escalation.

## Alternative dose titration in the medical therapy of acromegaly

### Increasing dose interval/decreasing frequency of administration of injections

**Figure 7:**
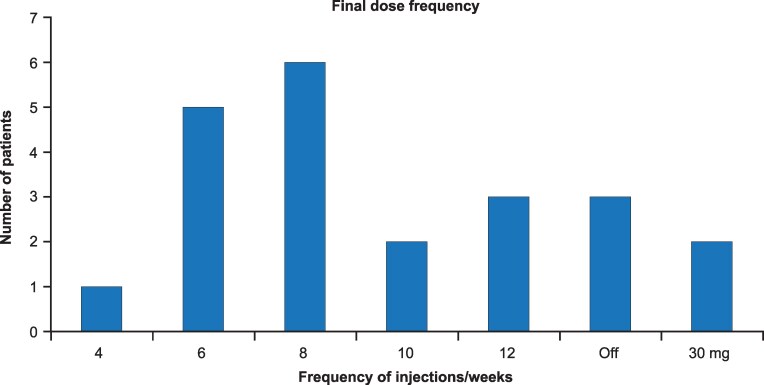
The final octreotide LAR 20 mg dosing interval in 22 patients enrolled in a clinical trial of systematic dose extension. Graph shows the final dose interval for all patients. If mGH was greater than 5 mU/L, then the dose of octreotide LAR was increased to 30 mg every 4 weeks. Reprinted from Turner et al (122) with permission from John Wiley & Sons. Abbreviations: LAR, long-acting release; mGH, mean growth hormone; Off, number of patients who did not require octreotide LAR treatment as mGH remained suppressed.

FDA-approved therapies include label guidance for dose titration except for CAM2029, an octreotide subcutaneous depot only approved in Europe at a single dose of 20 mg monthly (121). Lanreotide includes labeling for extended dosing as 120 mg every 6 or 8 weeks. Octreotide labeling goes up to 40 mg monthly. Outside of this label guidance, alternative dosing regimens for octreotide LAR, lanreotide depot, and pegvisomant have been studied with the goal of either decreasing medication burden and cost or improving acromegaly control. Several clinical trials have demonstrated successful use of up to 12 weeks extended dosing intervals (EDIs) for octreotide LAR and up to 8 weeks for lanreotide autogel (ATG) (122-126). When 22 patients with uncontrolled symptoms were initiated on octreotide LAR 20 mg every 4 weeks, and the dosing interval gradually extended based on biochemical parameters to up to 12 weeks (122), there was no difference in mean IGF-I between the 4-week and final dosing interval (Fig. 7). This strategy decreased the total number of injections and the cost of therapy. SOMACROL, an observational study, showed that an EDI for lanreotide 120 mg ATG was in active use in clinical practice in Spain and Portugal, with the most common EDI being 5 to 6 weeks with >90% biochemical control achieved in this cohort (127). EDI is not a viable strategy in all patients; it requires careful monitoring and prolonged active titration, as some patients require a return to 4-week dosing intervals to maintain biochemical and/or clinical control. While there are not enough data to guide EDI for pasireotide LAR, 1 retrospective study suggested that patients treated with 40-60 mg of pasireotide LAR may have their dose reduced to 20-40 mg without loss of biochemical control and associated improvement in blood glucose (128). Pegvisomant is approved for daily subcutaneous injections, but EDIs were considered given its half-life of 6 days. Indeed, several studies demonstrate the feasibility, acceptability, and reduced cost of once- or twice-weekly pegvisomant (129-131). While the total weekly dose may be similar between weekly and daily pegvisomant, some patients might be able to decrease their total weekly dose when pegvisomant is given as a single weekly injection vs a daily regimen (129, 130). This EDI for pegvisomant was effective both for monotherapy and in combination with SRLs, but combination therapy allows for lower weekly pegvisomant doses (131).

### Increasing SRL dose and/or frequency

Patients with symptoms uncontrolled on the maximal approved doses of octreotide LAR and lanreotide, higher-dose (octreotide, lanreotide), or higher-frequency (lanreotide) regimens have been studied in an attempt to improve control rates (132-134). Increased SRL exposure is safe, decreases GH/IGF-I, and increases the biochemical control rate. But, importantly, relatively few uncontrolled patients achieved complete biochemical control with this dose or frequency escalation. Therefore, most patients with symptoms uncontrolled on maximally approved SRL doses are likely to require an additional medication or alternative treatment modality. Treatment burden and cost, the need for tumor-directed therapy, and symptom control should be considered when pursuing these alternative dosing regimens.

In summary, despite improved diagnosis of acromegaly, there is an average duration from symptom onset to diagnosis of 5 years. Artificial intelligence has improved accuracy in facial recognition and learning models for earlier identification of the disease, but privacy, ethnic differences, and cost-effectiveness currently limit implementing these into general clinical practice. Algorithms that identify the likelihood of SRL responsiveness have advanced. However, the risk of overtreatment of patients inaccurately designated as SRL nonresponders and the need for standardization of the imaging and histologic diagnostic assessments limit their utility in real-world practice. The expanding choices for therapy may decrease treatment burden. Oral SRLs require a shorter time for dose adjustment and may minimize symptom fluctuation seen with long-acting formulations. More information is needed on medical therapy of acromegaly during preconception and pregnancy as well as on alternative strategies to individualize and optimize care, such as extending drug dose interval and the use of combination therapies.

## Data Availability

Data sharing is not applicable to this article as no data sets were generated or analyzed during the present study.

## Abbreviations

AIP: aryl hydrocarbon receptor–interacting protein
ATG: autogel
EDI: extended dosing interval
FDA: US Food and Drug Administration
GH: growth hormone
IGF-I: insulin-like growth factor 1
LAR: long-acting release
LC-MS: liquid chromatography–mass spectrometry
mGH: mean growth hormone
OGTT: oral glucose tolerance testing
PASQ: Patient-Assessed Acromegaly Symptom Questionnaire
SRL: somatostatin receptor ligand
SSTR2: somatostatin receptor 2
SSTR5: somatostatin receptor 5
US: United States
